# Research on the Micromorphology of the Native Surface of 2-Inch Aluminum Nitride Single Crystals

**DOI:** 10.3390/ma18051147

**Published:** 2025-03-04

**Authors:** Ruixian Yu, Gang Zhao, Kai Jiang, Wenjing Tang, Lei Zhang, Wei Xia

**Affiliations:** 1 School of Physics and Technology, University of Jinan, Jinan 250022, China; yuruixian0001@126.com (R.Y.); sps_zhaog@ujn.edu.cn (G.Z.); sps_jiangk@ujn.edu.cn (K.J.);; 2 Institute of Novel Semiconductors, State Key Laboratory of Crystal Materials, Shandong University, Jinan 250100, China

**Keywords:** two-inch AlN crystals, step-flow morphology, crystal domains, PVT

## Abstract

High-quality 2-inch aluminum nitride (AlN) crystals were grown using a double-zone resistance heating system, and the growth mechanism of AlN bulk crystals was further investigated. It was found that during the growth process, the vapor pressure at the growth interface, as well as the quality and structure of the seed crystal, was closely related to the growth conditions. The 2-inch AlN crystals were characterized using high-resolution X-ray diffraction (HRXRD) and optical microscopy. Optical microscopy observations of different regions on the native surface of the crystals revealed several morphologies, including regular step flow, irregular step flow, and domain-like structures. Comparisons showed that areas of the crystal surface with regular step-flow morphology exhibited high crystal quality, whereas the crystal quality decreased progressively as the step-flow morphology diminished. Therefore, the crystal quality can be preliminarily assessed through the surface morphology, providing guidance for improving the crystal growth process.

## 1. Introduction

Aluminum nitride (AlN) crystals, as ultra-wide bandgap semiconductor materials, have been widely applied across various fields due to their exceptional high-frequency power characteristics, excellent high-temperature stability, low energy loss, and outstanding ultraviolet transmission properties [1]. These applications include high-efficiency optoelectronic devices; high-power, high-frequency electronic devices; ultra-high-voltage power electronics; deep ultraviolet (DUV) early warning detection; and DUV LED-based disinfection [2,3,4,5]. In recent years, AlN has increasingly been applied in the field of nuclear technology, which involves demanding environmental conditions [6,7]. In addition, AlN crystals are considered ideal substrate materials for the epitaxial growth of aluminum-rich III-nitride alloys [8]. In the field of power electronics, AlN exhibits an exceptionally high critical breakdown electric field, which allows AlN-based power devices to achieve higher blocking voltage, ultra-low on-resistance, and ultra-fast switching speeds [9].

Although AlN crystals demonstrate outstanding performance, the full potential of their properties still depends on the quality of the crystals. Therefore, to fully utilize AlN crystals, a comprehensive understanding of their internal structure, crystal defects, growth mechanisms, and impurities is crucial. Many researchers have conducted systematic investigations into the growth characteristics of AlN [10,11,12,13].

Regarding growth morphology, Nagamatsu K et al. grew AlN crystal films using high-temperature metal–organic vapor phase epitaxy (MOVPE). The step-terrace structure of the AlN layer was found to be closely related to the off angle of the sapphire substrate [10]. Bryan I et al. investigated the relationship among crystal surface morphology, structural quality, impurity concentration, and growth temperature. The optimal growth conditions for high-quality (1$\bar{1}$00) AlN epitaxial layers have been established [11]. A surface dynamics framework for growing AlN crystals on the aluminum-polar face using the MOCVD method was further established, and the relationship between the surface morphology of films with different dislocation densities and step flows was investigated. The study results indicate that step morphology is influenced by the substrate orientation and the vapor pressure saturation level. By optimizing the growth conditions, high-quality crystal films with an ideal step morphology were successfully fabricated [12]. Qin Zuoyan et al. investigated the generation and evolution of subgrains during the PVT growth of AlN and proposed a suppression method to control the growth mode. They found that larger temperature fluctuations result in increased fluctuations in the supersaturation of gas-phase substances on the crystal surface, thereby enhancing the probability of forming high-index crystal faces [13].

The aforementioned studies on the morphology of AlN materials are based on AlN thin films. In this study, we successfully fabricated 2-inch, high-quality AlN crystals using a two-stage heated AlN growth system. The growth mechanism of AlN bulk crystals was further investigated, and the relationship between the native surface morphology and crystal quality was analyzed.

## 2. Description of AlN Crystal Growth Equipment

**Figure 1:**
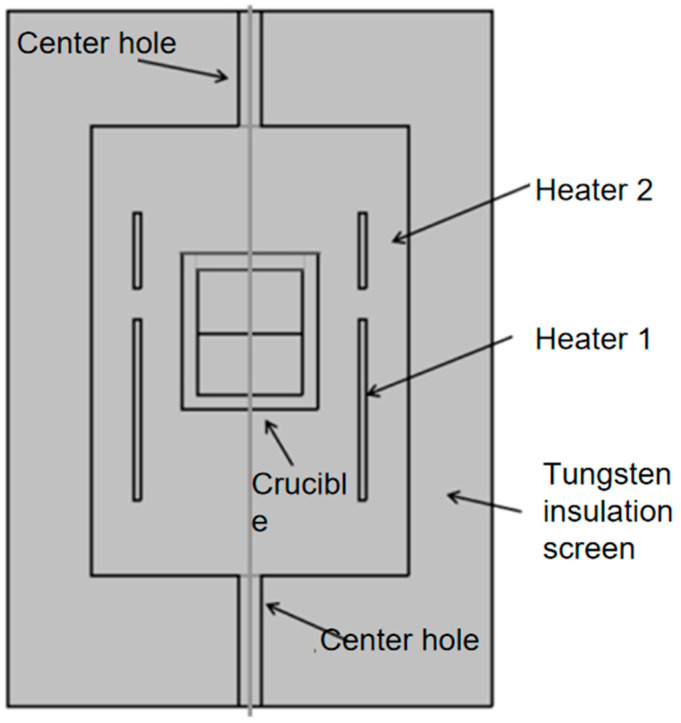
AlN growth system.

The growth system employed in this study is a self-developed two-zone resistive heating system, and the chamber structure is illustrated in Figure 1.

The primary function of the chamber is to maintain the vacuum environment and control the growth pressure necessary for the growth process. The tungsten heater supplies the heat source to the growth chamber, while the tungsten–molybdenum thermal insulation screen minimizes heat loss from the chamber. The AlN precursor is placed at the bottom of a sealed crucible, with the crystal growth occurring at the upper part. The heater and thermal insulation screens collaborate to provide the source temperature (2180–2400 °C) and a temperature gradient for the crucible. The temperatures of the upper and lower covers of the crucible are measured using an infrared thermometer. The growth of AlN crystals occurs in a nitrogen atmosphere.

The following parameters are fixed: heater 1 has a diameter of 130 mm and a height of 280 mm; heater 2 has a diameter of 130 mm and a height of 90 mm; crucible has an inner diameter of 90 mm, with a height of 140 mm.

The AlN crystal growth process primarily involves steps such as cleaning, heating, crystal growth, and cooling.

The chamber is evacuated with a vacuum pump to achieve a vacuum, followed by the introduction of high-purity N_2_ gas. This process is repeated 3 to 5 times. The oxygen, carbon, and water vapor impurities in the chamber are removed as completely as possible at room temperature.

Heating: The temperature is gradually increased to the growth temperature (2000~2300 °C). During this process, the chamber atmosphere is N_2_, with a pressure range of 70,000 Pa to 100,000 Pa. Impurities in the chamber are effectively removed during heating.

High-Temperature Growth: At the growth temperature, the chamber pressure is reduced to a range of 20,000 Pa to 70,000 Pa. The AlN precursor begins to decompose in the high-temperature zone, and the reaction gas is directed to the seed crystal at the top of the crucible, where crystallization takes place upon cooling. The primary reaction in this process is presented in Equation (1).

(1) $$AlN(s)⟺Al(g)+1/2N_{2}(g)$$

Cooling Process: Due to the relatively high temperature at the initial stage of cooling and the significant difference in the thermal expansion coefficients between AlN crystals and tungsten, the cooling process should be carried out as slowly as possible to minimize thermal stress within the crystal, thereby reducing the likelihood of crystal cracking.

Using this equipment, high-quality, 2-inch AlN crystals were successfully grown and 2-inch AlN wafers were obtained after subsequent processing, which included cutting, grinding, and polishing.

## 3. Discussion

### 3.1. Surface Dynamics of AlN Crystal Growth Using the PVT Method

**Figure 2:**
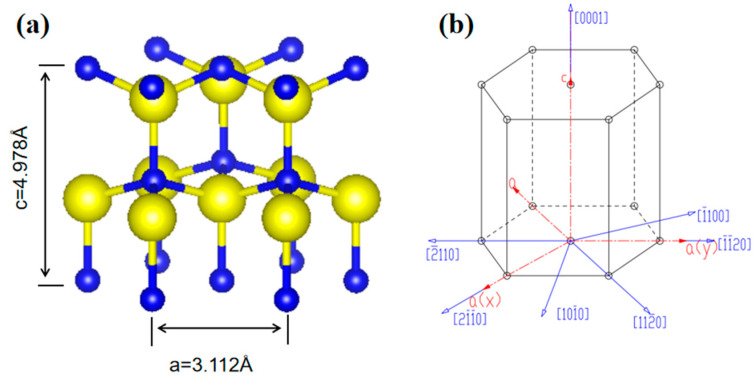
(**a**) The unit cell of AlN. (**b**) Crystal orientation index of AlN.

The stable phase of AlN crystals is the hexagonal wurtzite structure, which belongs to the P63 space group. The structure of AlN crystals and the commonly used crystallographic indices are shown in Figure 2. The fundamental structure of the AlN crystal is composed of Al-N_4_ tetrahedra, with each unit cell containing six Al atoms and six N atoms. The lattice parameters of the AlN crystal at room temperature are a = 3.111 × 10^−1^ nm and c = 4.978 × 10^−1^ nm. Under ideal conditions, the Al-N bond lengths in the AlN crystal are expected to be equal. However, in practice, the Al-N bond length along the [0001] direction differs from that along the [000$\bar{1}$] direction, resulting in a nonzero Al-N dipole moment vector that induces polarization. The cell parameters of AlN crystals, as obtained experimentally, may deviate from those of the ideal wurtzite structure, potentially influencing the properties of the crystals [14,15].

**Figure 3:**
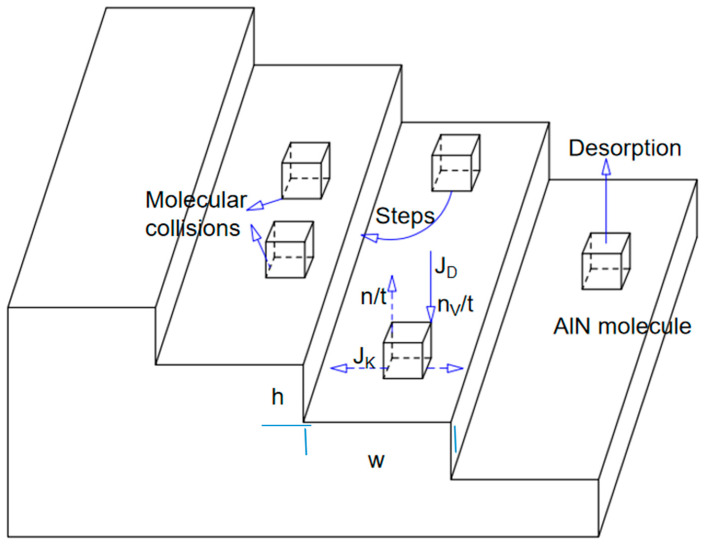
Schematic diagram of the movement of AlN molecules on the growth interface.

The quality of AlN crystals grown homoepitaxially via the PVT method is significantly influenced by the seed crystal quality. High-resolution X-ray diffraction (HRXRD) is commonly employed to evaluate the quality of seed crystals. A narrow full-width at half maximum (FWHM) of the (002) plane typically suggests superior seed crystal quality. A high-quality seed crystal provides suitable attachment sites for AlN molecules in the gas phase, thereby facilitating the transfer of structural information to the growing crystal. During the growth process, gas-phase molecules reaching the growth interface exhibit motion governed by the energy state of the interface. This molecular layer can be considered a thin film and is commonly referred to as the growth buffer layer. Molecules in the growth buffer layer may undergo the following processes: (1) Molecules migrate to step edges where the potential energy is minimized, form bonds with the crystallized lattice, and grow in alignment with the original crystal structure. (2) During motion, molecular collisions disturb the original structural information of the crystal, leading to two-dimensional nucleation. (3) Molecules detach from the growth interface, transitioning back to the gaseous state via desorption. Figure 3 illustrates the motion of AlN molecules at the growth interface. J_K_ denotes the diffusion flux of molecules within the buffer layer. J_D_ denotes the molecular arrival flux from the gas phase to the buffer layer. n denotes the density of molecules adsorbed within the buffer layer. t denotes the average molecular residence time within the buffer layer. w and h denote the width and the height of the crystal step, respectively.

This study aims to facilitate a more comprehensive analysis of the relationships among nucleation, steps, supersaturation, and molecular motion [12]. The following assumptions are proposed: the growth steps are considered ideal; gas-phase molecules are assumed not to nucleate on the steps; and the adsorption sites and energies at the step edges are sufficient to accommodate all adsorbed molecules. Hence, the net adsorption flux in the buffer layer is represented by Equation (2).

(2) $$J_{V}\left(x\right)=J_{D}-\frac{n(x)}{t}=\frac{\partial J_{K}(x)}{\partial x}$$

According to Einstein’s relation, the diffusion length LS in the buffer layer is related to the diffusion coefficient D and the average residence time t of molecules, as shown in Equation (3).

(3) $$L_{S}=\sqrt{Dt}$$

Since J_D_ is proportional to the gradient of n, Equation (4) can be derived.

(4) $$n\left(x\right)=J_{D}t+(n_{0}-J_{D}t)\frac{cosh(\frac{x}{L_{S}})}{cosh(\frac{W}{{2L}_{S}})}$$

Assume that x = 0 represents the center of the step, where the molecular adsorption density reaches its maximum. Under equilibrium growth conditions, the supersaturation in the buffer layer $\sigma_{s}(x)$ is expressed as the relative difference between n and n_0_, as described in Equation (5).

(5) $$\sigma_{s}\left(x\right)=\frac{n\left(x\right)-n_{0}}{n_{0}}=\left(\frac{J_{D}t}{n_{0}}-1\right)\left(1-\frac{cosh\left(\frac{x}{L_{S}}\right)}{cosh\left(\frac{w}{2L_{S}}\right)}\right)$$

With N_2_ as the background atmosphere, P_Al_ denotes the actual partial pressure of aluminum at the growth interface, while P_Al_^0^ represents the partial pressure of aluminum under thermodynamic equilibrium conditions. The relationship between the gas-phase supersaturation and the aluminum partial pressure is expressed in Equation (6).

(6) $$\sigma =\frac{P_{Al}-{P_{Al}}^{0}}{{P_{Al}}^{0}}$$

The saturated vapor pressure quantifies the deviation of the growth system from thermodynamic equilibrium and serves as the driving force for crystal growth. The following relationship can be derived from Equations (2), (5), and (6):

(7) $$J_{V}\left(x\right)=\frac{n_{0}}{t}\left(\sigma -\sigma_{s}\left(x\right)\right)$$

(8) $$\sigma_{s}\left(x\right)=\sigma \left(1-\frac{cosh\left(\frac{x}{L_{S}}\right)}{cosh\left(\frac{w}{2L_{S}}\right)}\right)$$

When x = 0, the saturated vapor pressure in the buffer layer $\sigma_{s}\left(x\right)$ reaches its maximum value, as shown in Equation (9).

(9) $$\sigma_{s,max}=\sigma \left(1-\frac{1}{cosh\left(\frac{w}{2L_{S}}\right)}\right)$$

The maximum saturated vapor pressure in the buffer layer ${(\sigma }_{s,max})$ is related to the actual partial pressure of Al at the growth interface (P_Al_), the width of the steps (w), the height of the steps (h), and the diffusion length (L_S_) within the buffer layer. In the early stages of growth, the step width (w) and height (h) are determined by the quality and structure of the seed crystal, while P_Al_ and L_S_ are influenced by the temperature, pressure, and other factors at the growth interface. The critical nucleation value is $\sigma_{s,2D}$(x). When $\sigma_{s}\left(x\right)>\sigma_{s,2D}$(x), crystal growth follows a two-dimensional nucleation mode. When $\sigma_{s}\left(x\right)<$ 0, the crystal will decompose. When ${0<\sigma }_{s}\left(x\right)<\sigma_{s,2D}(x)$, crystal growth proceeds according to the seed crystal step structure.

### 3.2. Analysis of the Surface Morphology of Naturally Grown AlN Crystals

The stacking sequence of Al and N atoms along the [0001] direction in hexagonal wurtzite AlN crystals is AaBbAaBb. Based on the surface dynamics of AlN crystal growth via the PVT method, the surface steps convey the seed crystal’s initial structural information for crystal growth. The adsorbed AlN molecules are captured at the step edges, thus facilitating crystal growth [16].

**Figure 4:**
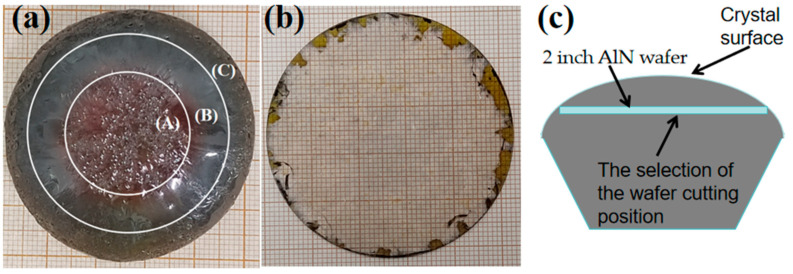
(**a**) Two-inch AlN crystal and (**b**) two-inch AlN wafer. (**c**) The selection of the wafer cutting position.

The lattice structure, surface energy, and atomic diffusion mechanisms of AlN, along with other factors, must be considered comprehensively during the growth process. The native morphology and quality of AlN crystals grown by the PVT method are closely related. Figure 4a shows a 2-inch AlN crystal grown by our research group, with the growth surface being the (0001) Al plane, and the surface is predominantly smooth. The center of the crystal (region A) features macro V-shaped pits, with sizes ranging from 0.5 mm to 1 mm. The surface at the edge of the crystal (region C) is relatively rough. Figure 4b shows a 2-inch (0001) AlN wafer obtained by machining at the position closest to the crystal surface. Figure 4c presents a side view of the AlN crystal, indicating the cutting position of the 2-inch wafer that is nearest to the crystal surface.

**Figure 5:**
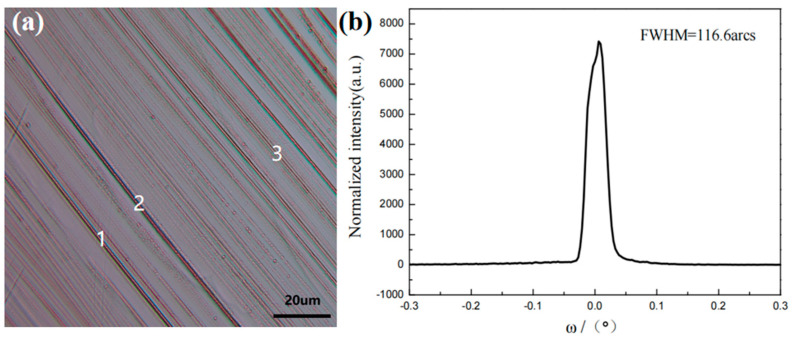
(**a**) Morphology of the step flow in the B region of the crystal under optical microscopy and (**b**) HRXRD rocking curve (002) of the B region of the wafer.

The optical microscope is used to observe different regions of the crystal’s native surface, followed by local characterization of the obtained wafer quality using high-resolution X-ray diffraction (HRXRD). The characterization points correspond to the observation points on the native surface of the crystal. Regular step flow was observed in the bright surface region (region B) between the center and edge of the crystal, with the step flow appearing as a set of parallel lines, as shown in Figure 5a. Since the growth information propagates along the direction perpendicular to the c-axis, uniform and regular steps are crucial for maintaining the quality of AlN crystals. Figure 5 shows that in most regions, the step width ranges from 3 to 6 μm, with smooth steps and minimal defects. In regions 1, 2, and 3, multiple steps are closely spaced, with step widths less than 3 μm.

The B region was characterized using high-resolution X-ray diffraction (HRXRD), as shown in Figure 5b. The half-width of the HRXRD rocking curve is 116.6 arcsec, with a single diffraction peak, indicating consistent crystal orientation and excellent crystallinity.

**Figure 6:**
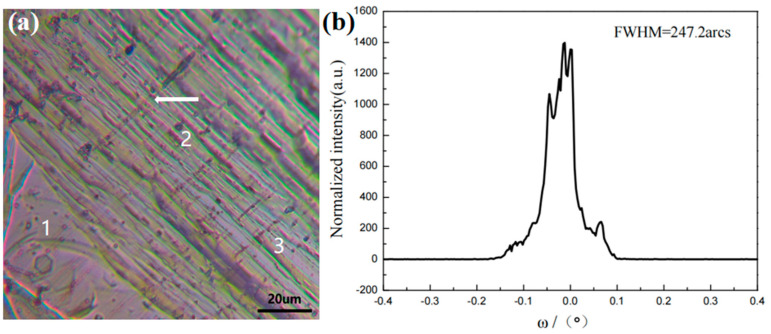
(**a**) Morphology of the step flow in the A region of the crystal under optical microscopy and (**b**) HRXRD rocking curve (002) of the A region of the wafer.

Points with relatively flat surfaces were selected in the central region (region A) and observed using an optical microscope. Figure 6a shows that the step growth in region 2 is relatively disordered, with a linear defect, indicated by the arrow, perpendicular to the growth steps. The morphology in region 3 begins to resemble a flat surface, with step widths exceeding 10 μm. Region 1 is a large, flat surface, and the entire A region is densely populated with numerous small polycrystalline particles.

The A region was characterized using high-resolution X-ray diffraction (HRXRD), as shown in Figure 6b. The half-width of the HRXRD rocking curve is 247.2 arcsec, with the diffraction peak showing additional peaks. It suggests a slight misalignment of the crystal orientation in this region, resulting in a degradation of crystal quality.

**Figure 7:**
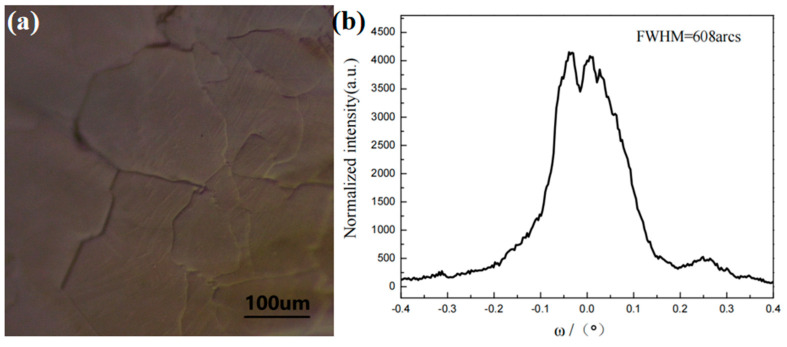
(**a**) Morphology of the step flow in the C region of the crystal under optical microscopy and (**b**) HRXRD rocking curve (002) of the C region of the wafer.

The edge of the crystal (region C) was observed, which is located next to the widening tungsten ring, with a relatively rough crystal surface. The expanded diameter of the tungsten ring serves to facilitate the gradual growth of bulk crystals along its sloped surface during the crystal growth process. The edge of the crystal with relatively flat regions was observed using an optical microscope, as shown in Figure 7a. The C region is composed of crystal domains with uneven morphologies and varying sizes. The macro photograph of the crystal (Figure 5a) clearly reveals visible holes in this region. Due to the large radial temperature gradient at the growth interface, the edges of the seed crystal experience ablation from the elevated temperature, leading to the formation of voids. The C region was characterized using high-resolution X-ray diffraction (HRXRD), as shown in Figure 7b. The half-peak width of the HRXRD rocking curve is 608 arcs, and the diffraction peak shows more pronounced splitting and an increased half-peak width, indicating further deterioration in the crystal quality of the C region.

The dynamical behavior of the growth interface is closely related to its microstructure. The microstructure of the crystal interface governs the growth mechanism, which subsequently dictates the dynamical laws governing crystal growth. Due to the lowest potential energy at the step edges, molecules diffuse toward the step edges, where a sufficient number of kink sites are available, providing positions for the molecules to arrange in an ordered manner. When the vapor pressure of the reaction atmosphere is too high, the gas molecules cannot be fully accommodated by the kink sites at the step edges. The existing growth information of the crystal will be disrupted, resulting in the introduction of defects during crystal growth. When step flow is absent on the growth surface, reactive gas molecules do not need to overcome the potential barriers caused by the steps and they continuously arrange themselves in an orderly manner along the lattice. The formation of crystal domains is due to the occurrence of local molecular misalignment during the alignment process. By comparison, the crystal quality is highest in regions with a uniform step-flow morphology on the native surface, while it decreases in regions with disordered step-flow morphology. The lowest crystal quality is found in the crystal domain region at the crystal edge. The crystal quality can be preliminarily assessed by observing the surface morphology, providing insights for improvements in crystal growth.

## 4. Conclusions

The relationship between the ordered growth of AlN molecules on steps and the saturated vapor pressure is explained using surface dynamics. The maximum saturated vapor pressure in the buffer layer $\left(\sigma_{s,max}\right)$ is related to the actual partial pressure of Al at the growth interface (P_Al_), the width of the steps (w), the height of the steps (h), and the diffusion length (L_S_) within the buffer layer. the w and h are determined by the quality and structure of the seed crystal, while the P_Al_ and L_S_ are influenced by the temperature, pressure, and other factors at the growth interface. The surface morphology of the crystal was observed using an optical microscope. A regular step-flow morphology was observed in the crystal smooth surface region. The half-width of the HRXRD rocking curve (002) in this region is 116.6 arcsec, with a single diffraction peak, indicating the highest crystal quality. Disordered step-flow morphology was observed in the crystal center region. The half-width of the HRXRD rocking curve (002) in this region is 247.2 arcsec, with additional peaks in the diffraction pattern. This suggests a slight misalignment of the crystal orientation in this region, leading to a degradation in crystal quality. Crystal domains were observed in the crystal edge region, with the half-width of the 002 HRXRD rocking curve measured at 608 arcsec. The splitting of the diffraction peak is more pronounced, indicating a lower crystal quality in this region. It can be inferred that the surface morphology of the crystal is closely related to its quality. The crystal quality can be preliminarily assessed based on its surface morphology, providing directions for improvements in crystal growth.

## Data Availability

The original contributions presented in this study are included in the article. Further inquiries can be directed to the corresponding authors.
